# Political philosophy of experimentation

**DOI:** 10.1007/s13194-025-00665-1

**Published:** 2025-06-17

**Authors:** Massimiliano Simons

**Affiliations:** Department of Philosophy, Maastricht University, Maastricht, The Netherlands

**Keywords:** Philosophy of experimentation, Political philosophy, Joseph rouse, Hans radder, Gilbert Hottois, Jerome Ravetz

## Abstract

In this article, I sketch out the contours of a political philosophy of experimentation. Drawing on the work of Joseph Rouse, Hans Radder, Gilbert Hottois, and Jerome Ravetz, I argue that such a political philosophy of experimentation already exists, but has often been rendered invisible. Mainstream Anglophone philosophy of science often focuses on epistemological questions, typically at the expense of political questions about science. I argue that such a politicization of philosophy of science can be linked to the topic of experimentation, which transforms epistemological questions about representations of the world into political questions about interventions in the world. In the first section of the article, I examine the work of a number of political philosophers of experimentation in order to make explicit what I mean by a “political” philosophy of experimentation. In the second section of the article, I present a systematized argument why a philosophy of experimentation should be political. Finally, in the last section, I map out more explicitly the set of questions that are central to such a political philosophy of experimentation, in particular questions about what I call the politics of interaction, referring to how science and society should interact; the politics of organization, referring to how science should be organized; and the philosopher as political activist, referring to the question of whether philosophers should also act on their theories.

“if science is technoscience, it inevitably raises practical questions.” (Hottois, 2018, p. 123).

## Introduction

A philosophy of science that systematically reflects on experimentation will transform itself into a political-philosophical project. This transformation is not only possible, but has already taken place. These are the two central claims of this article. First, I will argue for the second claim by a review of the work of some philosophers of experimentation (Sect. 2). In particular, I will use their work to explore the different possible meanings of ‘political’ philosophy of experimentation. In fact, there are at least four possible ways in which a philosophy of experimentation can be political: it can be political either in its motivation or in its effects (2.1.), in the project of a philosophy of experimentation (2.2.), and in the (political) actions of a philosopher of experimentation (2.3.).

Based on this literature review, the next section will articulate in a more systematic way what constitutes a political philosophy of experimentation (Sect. 3). Specifically, I argue that a philosophy of science that reconceptualizes science as an intervening activity, rather than as a mere representation of reality, transforms science from a purely epistemic activity into one that is also political. This politicization redirects the attention of philosophers to different political-philosophical topics about science, which I will explore in Sect. 4. In addition to the usual ethical questions about science and technology (4.1.), there are at least three major political issues at stake: an examination and evaluation of the structures through which scientific practices shape society (what I will call the *politics of interaction*) (4.2.); an examination and evaluation of the social organization of these scientific practices (what I will call the *politics of organization*) (4.3.); and the practical question of whether and how philosophers should themselves be politically active (what I will call the *philosopher as activist*) (4.4.).

Let me begin by emphasizing a number of qualifications. First, the claim of this paper is not that political philosophy of experimentation is the *only* field doing this kind of political-philosophical work. On the contrary, other traditions have engaged in very similar projects. For example, much of the work in Science and Technology Studies (STS) is concerned with these political questions. Similarly, one could point to the political sociology of science (Blume, 1974; Frickel & Moore, 2006) or political epistemology (Vogelmann, 2024). The existence of this literature does not negate my argument, which is to show that these questions are *also* central to a philosophy of experimentation. The claim is not that these issues have been generally neglected, but that they are typically not seen as part of the field of philosophy of science - and that this claim is unjustified. There is no intrinsic reason why such a political philosophy of experimentation cannot be seen as a philosophy of science proper.

Second, of course, there have also been philosophers of science who have done political-philosophical work. Numerous studies have drawn on feminism and postcolonialism to argue that scientific practices are often fraught with problems of racism, sexism, ableism, and so on (e.g. Harding, 2011). Similarly, recent work on ‘values in science’ has emphasized the role of non-epistemic values, including political ones, in scientific practices (Longino, 1990; Douglas, 2009; Elliott, 2022). My argument is not meant to negate this work. Indeed, many of its arguments can be translated into similar political arguments about experimentation. However, this often implies forms of translation. There is a tendency to frame political issues about science in terms of scientists’ beliefs and values. As we shall see, the aim of this paper is to show how a focus on experimentation politicizes scientific practices in different but complementary ways.

## What is ‘political’ about political philosophy of experimentation?

This section provides a selective overview of how philosophers of experimentation have engaged with political issues in their work. I mobilize these authors to highlight the different ways in which a philosophy of experimentation can be political. This is important in order to make clear the sense in which this article will flesh out a political philosophy of experimentation. I am not so much concerned with the artificial application of theories from political philosophy to issues in the philosophy of science (see Rouse, 1987, epilogue). Rather, I am interested in how a philosophy of science that reflects on experimentation becomes a political project *from within*. However, such an internal political dimension of philosophy of science can refer to at least four things: the *motivation*, the *effects*, the *project* of a philosophy of experimentation, and the *political actions* of a philosopher of experimentation.

### Political motivations and effects

Let me begin by discussing the first two possible meanings of a political philosophy of experimentation, which I will treat together because I think they are the least interesting for my argument: a philosophy of experimentation can be political either in its motivation or its effects.

By *motivation* I mean the reason(s) why a philosopher of science takes experimentation as an object of analysis. It is not uncommon for a philosopher to do so because of a politically inspired motives. A good example of this is Harry Collins’ *Changing Order* (1985). The book itself consists of a series of case studies that start from the assumption that science is “a cultural activity” and not a “locus of certain knowledge” (1985, 1). The central claim is that there is what Collins calls an ‘experimenters’ regress’ in the replication of scientific findings: “since experimentation is a matter of skilful practice, it can never be clear whether a second experiment has been done sufficiently well to count as a check on the results of a first. Some further test is needed to test the quality of the experiment– and so forth.” (1985, 2).

Throughout the book, Collins makes his case in standard epistemological fashion. But then there is a short postscript that aims to explain its broader political relevance. This redescription of science as skillful practice seems to serve as a critique of what Collins calls the “algorithmical model” which “encourages the view that the formalized accounts of scientific work found in the journals are complete accounts” (1985, 159). This falsely promotes the image of science as complete certainty that you either accept or reject. In contrast, Collins wants to promote an “enculturational model”, where the “locus of knowledge is not the written word or symbol but the community of expert practitioners” (1985, 159). For Collins, what is at stake is a different appreciation of the political role of scientific experts: “Science, however, is not a profession that can take from our shoulders the burden of political, legal, moral and technological decision making. It can only offer the best advice that there is to be had. To ask for more than this is to risk widespread disillusion with science with all its devastating consequences.” (1985, 167) Thus, it could be argued that Collins’ philosophy of experimentation is political because its epistemological thesis is motivated by political concerns about the role of experts in society.

Similarly, a philosophy of experimentation can claim to be political because of the (un)intended political *effects* of its proposed analysis. For example, the work of Latour (1983, 1987) could be read as a (re)description of science that focuses on the role of experimentation, instruments and technology. It emphasizes how scientific facts are always constructed within a network of actors and never stand alone. According to some, this epistemological argument risks having the political effect of promoting a skeptical argument about science, undermining its authority (e.g. McIntyre, 2018). Of course, this may not have been Latour’s intention, but it may well have been the effect.

We find a different story about the *intended* effects of his work in his magnum opus *An Inquiry into Modes of Existence* (2013). Here, Bruno Latour opens his argument with an anecdote about “some fifteen French industrialists responsible for sustainable development in various companies, facing a professor of climatology, a researcher from the Collège de France.” (2013, 2) One of these industrialists raises the question: Why should we believe you, more than the climate sceptics? To Latour’s surprise, the climatologist’s answer does not appeal to the rationality of his arguments. Instead, he begins

to lay out before his audience the large number of researchers involved in climate analysis, the complex system for verifying data, the articles and reports, the principle of peer evaluation, the vast network of weather stations, floating weather buoys, satellites, and computers that ensure the flow of information […]. ‘And, in the other camp,’ he adds, ’what do we find? No competent researcher in the field who has the appropriate equipment’. (Latour, 2013, p. 3)

In other words, to counter the skeptic, the scientist invokes the network of instruments and actors needed to study climate change. It is therefore possible to read Latour’s philosophy of experimentation as political in this sense: it aims to have certain political effects, especially on debates about climate skepticism.

What these cases of political *motivation* and *effects* have in common, however, is that the actual work of the philosopher of science remains primarily epistemological, and thus business as usual. In other words, they do not engage directly with a political argument in their analysis. Instead, the fundamental questions at stake in these projects remain questions better labeled epistemological. A novel story about how scientists produce knowledge may be a pawn in the broader political game of Collins or Latour, but it remains seemingly apolitical in itself. In contrast, what I call the ‘political philosophy of experimentation’ concerns cases where a focus on experimentation is incompatible with business as usual. Instead, a focus on experimentation transforms the philosophical *project* of philosophy of science into something closer to what one would recognize as political philosophy (see 2.2.).

There are a number of objections to the way I have framed the issue so far. Some readers may question my reading of Collins and Latour as apolitical. Is Collins’ recent work on expertise (Collins & Evans, 2007) or Latour’s work on climate change (Latour, 2004) not clearly political? This is true, but my response is twofold. First, in both cases, this political project was largely invisible in their early work, hidden behind the ambition to simply describe how science works. In other words, there was at least the impression that one could deal only with epistemological questions about science without having to engage in political philosophy. Only in separate, later work did they attempt to translate this explicitly into political issues. Second, it is precisely because of this explicit political ambition that these later works are typically not classified as philosophy of science. It is against this boundary work that I argue here, boundary work to which Collins and Latour contributed (unintentionally) by keeping political philosophy out of the official picture of their early work.

But this answer might lead to another objection, namely about the usefulness of the distinction between epistemology and political philosophy. It is possible to read authors such as Collins and Latour as attempting to problematize such a distinction between science and politics. This is, for instance, the main concern of Steven Shapin and Simon Schaffer’s book *Leviathan and the Air-pump* (1985). The book aims to explore “the historical circumstances in which experiment as a systematic means of generating natural knowledge arose, in which experimental practices became institutionalized, and in which experimentally produced matters of fact were made into the foundations of what counted as proper scientific knowledge.” (Shapin & Schaffer, 1985, p. 3) The conclusion is that the epistemic standard of experimentation is itself a product of a broader political debate about the order of society: “the problem of generating and protecting knowledge is a problem in politics, and, conversely, […] the problem of political order always involves solutions to the problem of knowledge.” (Shapin & Schaffer, 1985, p. 21) Thus it seems that reflection on politics and science always go hand in hand.

This distinction is indeed problematic, but the distinction between politics and science is not the same as the distinction between political philosophy and epistemology. Even if one recognizes that science is always political, this does not in itself transform the philosopher of science from an epistemologist into a political philosopher. Although Latour, Collins, and Shapin and Schaffer suggest a picture in which experimentation is always linked to the political, they are still primarily concerned with developing an epistemology rather than a political critique of science. What I am looking for is not just an epistemology of science that takes the political into account (a philosophy of *politicized* science), but the possibility of a more direct political critique of science itself (a *politicized* philosophy of science). The problem is not that these authors do not allow for this, but that they typically do not really do it.

### The political project

There are, however, politicized philosophers of experimentation. For them, philosophy of experimentation is political as a *project*. But they are not to be found amongst the most frequently mentioned philosophers of experimentation such as Hacking (1983), Franklin (1986) or Galison (1987). Instead, they are usually left out of the picture, such as Rouse (1987), Radder (2012), Hottois (1979) or Ravetz (1971). Indeed, their lesser fame within philosophy of science might even be related to the politicization of their philosophies of experimentation. They may be less frequently cited within philosophy of science because the way in which they proposed to transform philosophy of science into a political *project* partly alienated them from mainstream philosophy of science. Instead, they are regularly reclassified as ethicists, STS scholars, or philosophers of technology. The aim of this paper is to bring this hidden tradition back into the limelight.^1^

Take the example of Joseph Rouse, who focuses explicitly on experimentation in his book *Knowledge and Power: Toward a Political Philosophy of Science* (1987). As he explains in the book’s preface, his overall project is to understand “science as a field of practical activity”, emphasizing the role of experimentation in science, which in turn leads him to argue that:

We cannot readily separate the epistemological and political dimensions of the sciences: the very practices that account for the growth of scientific knowledge must also be understood in political terms as power relations that traverse the sciences themselves and that have a powerful impact on our other practices and institutions and ultimately upon our understanding of ourselves. (Rouse, 1987, xi)

These claims may seem reminiscent of what we have seen in Shapin and Schaffer (1985). But Rouse mobilizes these claims to explicitly raise political-philosophical questions about science. Here, philosophy of experimentation becomes political as a *project.* “We must ask how the natural sciences have changed us and in what terms we can best critically understand and assess these changes.” (1987, x) In particular, Rouse attempts to reconceptualize the concept of power, following Foucault (1975), as something that always goes hand in hand with knowledge: we can only know things by controlling them, and by controlling them, we gain knowledge about them. “Technical control, the *power* to intervene in and manipulate natural events, is not the application of antecedent knowledge but the form scientific knowledge now predominantly takes.” (Rouse, 1987, p. 20) A critique of science is therefore always a critique of what control the sciences (should) have over the world and over us.

A second example is Hans Radder, who in his 1984 dissertation develops a philosophy of experimentation that analyzes not only the role of theory in science, but also that of its ‘material realization’: “I wish to draw explicit attention to experimental natural science as a *material production process*: experiments need not only be interpreted theoretically, but are also realized materially.” (Radder, 2012, 67).^2^ With ‘material realization’, Radder wants to emphasize how all scientific theories, if they are to be investigated in scientific practices, have to be materially realized in a material and social setup in the laboratory (in the form of experiments, instruments, etc.). For Radder, this does not mean that theory plays no role in science. The materialization of science is always complemented by the *nonlocal* meanings of concepts: although concepts derive from materially and locally realized experiments, they also abstract from these local conditions and can be applied in other contexts. But this requires work that allows one to argue that the same (nonlocal) concept can be realized through different material realizations: “the notion of nonlocality entails that the meaning of scientific and technological practices, processes, and products is neither fully local nor simply universal. This meaning transcends the separate local contexts in a nontrivial way, but its scope is bounded by contingent historical developments.” (Radder, 1996, p. 3)

This framework allows Radder to transform his philosophy of experimentation into a political *project*. To be useful, scientific theories must be embedded in institutional, economic, and technological structures that determine what counts as valid scientific inquiry. Radder therefore explored how “scientific or technological knowledge and social power are intrinsically related to each other” (1986, 665). His argument is rather straightforward: “Technology is only (can only be) instrumentally successful, if certain specific material *and* social conditions are fulfilled; and an assessment of technology therefore not only concerns its effects but also and especially its conditions.” (Radder, 1986, p. 678) These conditions, Radder continues, include the social and political conditions that make an experimental or technological system work, and on which all scientific practices are based. This makes the material realization of scientific systems by definition a political matter.

Radder (2019) has translated this into a political critique of the “commodification” of science. Here he draws on his earlier philosophy of science: it is this play between material realization and nonlocal meaning that allows him to treat commodification not as an abstract distortion of pure research, but as a concrete reconfiguration of experimental practices shaped by market forces and political interests. For example, Radder is highly critical of current patenting practices, which often overlook a crucial distinction between patenting a *process* and patenting a *product*. A particular molecule, genetically modified organism or experimental effect must always be materially realized, but can typically be obtained in different ways (i.e. the concept behind it has non-local meaning). However, patents are typically linked to a (non-local, often ideal) product rather than a (materially realized) process. This can inhibit attempts to obtain the same product in more efficient ways, since all such alternative ways are also covered by the patent.

A third example is that of Gilbert Hottois (see Simons, 2025). In his dissertation, Hottois (1979) argues that science is more than symbols, and has an ‘operational’ side: science does not just describe the world, but is a way of operating on the world. Through both experimental and mathematical techniques, science allows us to develop operations to change and reshape the world. In order to re-emphasize this dimension, Hottois has been promoting the concept of *technoscience* since the 1970s (Hottois, 2018). This reconceptualization of science as technoscience leads to a shift in Hottois’s philosophy of science from epistemology to political philosophy. Hottois often frames this shift in Kantian terms: once science is reconceptualized as technoscience, it shifts from being a problem of pure reason to a problem of practical reason: “if knowledge, at every level, is synonymous with *power* and *action*, philosophical reflection on science is less a matter of pure Reason than of practical Reason” (Hottois, 1988, p. 10).^3^

Once we understand technoscience as operationality, philosophy of science is not primarily concerned with evaluating whether scientific findings are correct or not, but with evaluating the interventions and changes that technoscience brings to society. Whereas traditional philosophy of science asks the question ‘Is this an accurate representation of the world?’, a philosophy of technoscience asks a different question: ‘Do we want to live in this newly constructed world?’

In answering this question, Hottois is reluctant to impose any a priori restrictions on technoscience. This reluctance is a product of his broader philosophy of technology, which fundamentally associates technology with creative openness and unpredictability. This, in turn, is a product of his underlying worldview, which interprets the world as a dynamic set of processes: the world is constantly changing, constantly creating new ways of being, and technoscience is the mere extension of this.

Hottois often illustrates this with the concept of ‘prehistory’. Prehistory, which refers to the period before any written historical record existed, is fundamentally characterized by change and contingency. Change, because it is a history of the emergence of the cosmos and of life on Earth. Contingency, because there is no intrinsic reason why humanity should be the product of this process. By analogy, Hottois speaks of ‘posthistory’: the future is also characterized by change and contingency. There is no intrinsic reason why humanity will survive in the long run, let alone in its present form:

The evolutionist perspective tends to see history as an extraordinarily short, undeniably specifically human moment, preceded by an extraordinarily long prehistory and possibly followed by a ‘posthistorical’ evolution, of which we can anticipate nothing, because the future is felt today to be both open (the indefiniteness of the possible) and opaque (the impenetrability of any anticipation once we move even a few decades away from the present). (Hottois, 1988, 92–93)

For Hottois, a philosophy of technoscience embraces this dynamic worldview and recognizes that our technoscientific society is fundamentally subject to opaque change and contingency. Any attempt to impose a set of timeless ethical norms on technoscience runs the risk of projecting contingent moral standards onto human posthistory.

But Hottois also does not want to argue that every technoscientific intervention should be allowed. Rather, he wants to institutionalize and practice the ‘guidance’ (*accompagnement*) of technoscience: a stance that respects the openness and opacity of technoscientific development, but also constrains this process in a pragmatic way. Although there are no ahistorical ethical norms to which technoscience can be subjected, each society has its own set of (evolving) norms. The question then becomes how to manage these two temporal processes together without one completely disrupting the other. This is why Hottois’s *project* is political: it does not aim to prescribe ethical norms, but to politically organize a ‘philosophical guidance’ of technological development through societal norms.

The last example is Jerome Ravetz. In *Scientific Knowledge and Its Social Problems* (1971) he formulates a critique of the ‘industrialization of science’. Throughout the twentieth century, science has moved from Little Science to Big Science (Solla Price, 1963), the latter being inextricably linked to economic and industrial factors: “Applied science has now become the basic means of production in a modern economy.” (Ravetz, 1971, p. 21) For Ravetz traditional philosophy of science is unable to conceptualize this transition. Its categories and problems derive from an earlier time, when science had to struggle with religion and other social institutions for epistemic authority. But for Ravetz, “the heavy warfare with ‘theology and metaphysics’ is over.” (Ravetz, 1971, p. 20) In the twentieth century, the problems of scientific practice are mainly related to the social organization of science. “At the present time, the deepest problems in the understanding of science are social rather than epistemological. The older problems of the attainment of truth or its substitute, have given way to the concern for the maintenance of the health of science, and for the control of its applications.” (Ravetz, 1971, p. 71).

In his alternative philosophy of science, Ravetz emphasizes the experimental side of scientific practices: “For a comparison of the present age with the one preceding it, we can best start with the changed technical character of the work of scientific research; for from this follow the changes in its social institutions, and social practices.” (Ravetz, 1971, p. 44) Science is thus understood primarily as a *craft activity*, full of implicit knowledge that is difficult to articulate to outsiders. Ravetz draws heavily on Michael Polanyi’s (1958) notion of ‘tacit knowledge’: the process of scientific knowledge cannot be formalized, but always remains a craft to which one can at best be initiated to by insiders.

From this perspective, the challenge of industrialization becomes clear. It risks destabilizing this precarious scientific craftwork by imposing external formal rules on the scientific community, either by the state or by industry. The industrialization of science leads to four forms of problematic scientific activities: “shoddy science, entrepreneurial science, reckless science, and dirty science” (Ravetz, 1971, p. 57). As Steven Shapin summarizes in his review of the book, this concerns “stuff no-one cares about [but fills CVs]; stuff done solely for the sake of grants and jobs; stuff done without adequate reflection on its consequences for the wider society; and stuff whose end-product ‘lies beyond the pale of civilized practice and morality’ - for example, science needed for the production of atomic, biological, and chemical weapons” (Shapin, 1997, p. 341). In all these cases, the problem is the same: external criteria disrupt the internal criteria of adequacy associated with the tacit craftsmanship of science.

The *project* of Ravetz’ philosophy of experimentation thus becomes clearly political: it aims at a political critique of these destructive tendencies in contemporary science. But it also becomes the cultivation of an alternative way of doing science, which he calls ‘critical science’. In critical science, “collaborative research of the highest quality is done, as part of practical projects involving the discovery, analysis and criticism of the different sorts of damage inflicted on man and nature by runaway technology, followed by their public exposure and campaigns for their abolition.” (Ravetz, 1971,. 424) Ravetz is thinking of scientists who map the risks of pollution, genetic modification or nuclear power. His hope is that when this critical science “becomes incorporated into a coherent philosophy of science, it will provide the basis for a transformation of scientific inquiry as deep as that which occurred in early modern Europe.” (Ravetz, 1971, 428–429) Crucial to this critical science is “an awareness of craft skills at all levels, and the conscious effort of mastering new skills.” (Ravetz, 1971, p. 429).

### The political actions of the philosopher

In the previous section I discussed four examples of philosophers of experimentation whose *projects* become political. I paid particular attention to Hottois and Ravetz because their work also adds an additional dimension to the political philosophy of experimentation. In addition to formulating a political critique of science, they also translate their philosophical projects into direct *political action*. A political philosophy of experimentation can thus go one step further: the philosopher becomes an activist.

Hottois, for example, saw the ‘philosophical guidance’ of technoscience embodied in the emerging field of bioethics, and in particular in bioethics committees (Hottois, 2004). Bioethics committees steer new technoscientific developments, but do so without recourse to fundamental ethical principles: “Scientific-technical knowledge and symbolic knowledge can interact there without domination.” (Hottois, 2005, p. 29) Instead, their composition is often pluralistic, with members from very different ideological backgrounds. Moreover, their proposals are usually framed as provisional decisions that can be revised when future committees revisit the issue.

However, Hottois not only described these bioethics committees as an illustration of the guidance of technoscience, but also *participated* in the creation and practice of these novel institutions. In 1987, for example, he participated in the Belgian national conference on “Bioethics in the 1990s” (Demeester, 1987) as a counterweight to the otherwise dominant Catholic voices. Together with Charles Susanne, he also founded the Center for Interdisciplinary Research in Bioethics (CriB) at the Free University of Brussels (ULB), and was an active member of the Belgian National Committee on Bioethics from 1996 to 2001, as well as of the European Group on the Ethics of Science and New Technologies (GEE) of the European Commission. This work also led to the publication of several encyclopedias of bioethics (Hottois & Parizeau, 1993; Hottois & Mizza 2001).

Similarly, Ravetz spent his career trying to *practice* his ‘critical science’. In fact, he was already doing this when he wrote his book in 1971, as a member of the British Society for Social Responsibility of Science (BSSRS). The BSSRS aimed to put the question of scientists’ responsibility for their inventions and discoveries on the political agenda. Ravetz was also Executive Secretary of the Council for Science and Society (CSS) from 1973 to 1976. The CSS was a non-profit organization established in 1973 to study the social effects of science and technology, with a particular focus on the social responsibility of the scientist. Within the Council, Ravetz was responsible for drafting the report ‘The Acceptability of Risks’ and was a member of the Genetic Manipulation Advisory Group (1977–1978).

From this activist perspective, we can also make sense of two ideas often associated with Ravetz’s work: *NUSAP* and *post-normal science*. NUSAP refers to a notational system that Ravetz developed with Silvio Funtowicz to provide a way of communicating the level of uncertainty in science in policy debates (Funtowicz & Ravetz, 1986, 1987). NUSAP stands for *Numeral*, *Unit*, *Spread*, *Assessment* and *Pedigree*. For instance, if we want to be informed about the environmental risk of a new chemical product, we are likely to be given a quantitative figure, a percentage, to express the risk. This figure alone tells us very little. It is meaningless without the body of tacit knowledge that the scientist typically uses to interpret it. The NUSAP system is one way of compensating for this lack of tacit knowledge, which is not available to policy makers, other scientific disciplines or the general public. “We therefore need a new means of evaluating scientific work [in situations where this tacit knowledge of quality is lacking] which should provide clear, explicit and public guidelines for analysis and communication, while retaining the skills and judgments of the best traditional scientific practice.” (Funtowicz & Ravetz, 1987, 412–413) Thus, while *Numeral* refers to the number; *Unit* refers to the unit of measurement (e.g. parts per million, ppm); *Spread* refers to the margin of error associated with that number; *Assessment* in turn creates space to introduce some qualitative judgements associated with the number (e.g. whether this is a conservative or optimistic prediction); and *Pedigree* refers to a general evaluative description of how this number was generated and how it should be used.

A similar reading can be given to Funtowicz and Ravetz’ notion of post-normal science. The concept refers to Thomas Kuhn’s (1970) notion of normal science, but argues that we are now dealing with scientific facts that no longer follow Kuhn’s model. Instead of dealing with scientific facts within a scientific community, we are dealing with scientific facts within society, where the stakes are high and the level of certainty is low. “We call it ‘post-normal’ to indicate that the puzzle-solving exercises of normal science (in the Kuhnian sense) which were so successfully extended from the laboratory to the conquest of Nature, are no longer appropriate for the resolution of policy issues of risks and the environment.” (Funtowicz & Ravetz, 1993, p. 750).

A particularly influential idea associated with post-normal science is Funtowicz and Ravetz’s plea for ‘extended peer communities’: since the impact of scientific knowledge is not confined to the laboratory and scientists, but extends to society at large, other stakeholders should have a say in the process. “The dynamic of resolution of policy issues in post-normal science involves the inclusion of an ever-growing set of legitimate participants in the process of quality assurance of the scientific inputs.” (Funtowicz & Ravetz, 1993, p. 752) The motivation is not only political, based on the idea that those affected should have a say. The motivation is also epistemic: since the other stakeholders are affected, they also have potentially privileged access to other forms of knowledge, linked to their local contexts, that are crucial for a proper assessment of the technology in question: “an extension of peer communities, with the corresponding extension of facts, is necessary for the effectiveness of science in meeting the new challenges of global environmental problems.” (Funtowicz & Ravetz, 1993, 754–755)^4^

In more recent work, Ravetz has similarly mobilized his work on post-normal science and NUSAP to issues as diverse as sustainability (Ravetz, 2006), GMOs (Ravetz et al., 2013) and COVID19 (Saltelli et al., 2020).

## Sketch of a political philosophy of experimentation

In this section, I aim to extrapolate a more systematic argument in favor of a political philosophy of experimentation. The argument will consist of three steps. The first step is the claim that scientific practices should be understood not (only) as representation, but (also) as intervention. This leads to the second step, namely that scientific practices interpreted as interventions provoke political-philosophical questions. The concern is no longer only epistemic values, but also moral and political values, and even more directly political questions of power and control. This makes the philosophy of experimentation a political project. The third step is then to articulate the specific political questions that arise from this transformation, which will be explored in Sect. 4.

### Scientific practices as intervention

As we have seen in the previous examples, the starting point for the philosophy of experimentation is a different image of science. Echoing Ian Hacking’s (1983) famous book, science is not about *representing* but about *intervening*. Some might object that this claim is too strong, since Hacking’s book speaks of representing *and* intervening, whereas I present it here as an exclusive disjunction. Is it not preferable to say that some parts of science are best understood as descriptive, while other parts are best understood as intervening? I think the points below still hold, even if we accept this weaker version of the argument. Even if only parts of science intervene, we still need a philosophy to deal with those parts. And the argument would then be that, at least for those parts, a political philosophy of experimentation is justified.

However, philosophers of experimentation tend to make the stronger claim that a philosophy of experimentation is not just about adding experimental practices to the traditional picture of science, which focuses on theoretical practices. Rather, the philosophy of experimentation aims to overcome such a false dichotomy. This is clearly emphasized by Rouse: “Even theory is more practical and craftlike than philosophers usually recognize. Thus, the problem is not the neglect of one part of science (experiment) in favor of another (theory), but rather a distorting perspective on the scientific enterprise as a whole.” (Rouse, 1987, xi) In other words, theories and representations still have a role to play, but their role should be redefined as forms of intervention.

If we accept that scientific practices are best understood as forms of intervention, what exactly do we mean by ‘intervention’? At its core, the idea—central to many philosophers of experimentation—is that scientific inquiry necessarily involves transforming aspects of the environment. Through the use of experiments, instruments, and technologies, scientists actively shape the conditions under which knowledge is produced. These interventions are essential for constructing systems that can reliably understand and predict the world.

More precisely, the point is this: scientific knowledge is fundamentally shaped by its development and testing in controlled environments—most notably, the laboratory. Even in fieldwork, where conditions are less easily regulated, researchers often strive for partial control. A philosophy of experimentation must therefore center on this dimension of *control*. Such control is not passive; it involves active intervention, a technical manipulation of reality. This often entails the construction of conditions or circumstances that did not previously exist, or the deliberate generation of what Hacking (1983, 220) famously called “novel phenomena”—entities or processes that do not occur naturally, or occur rarely and weakly. In this sense, scientific knowledge emerges within, and is dependent upon, an at least partly artificial environment. It is this artificiality that a philosophy of experimentation must foreground: to understand how scientific theories are produced, we must attend to the interventions that create the conditions under which epistemic inquiry becomes possible.

But the argument becomes particularly interesting and provocative when we turn to the question of how scientific knowledge produced in an artificial environment can subsequently be applied to other environments. Philosophers of experimentation argue that this process must be accompanied by a set of similar, though not necessarily identical, measures to control these novel environments. The most radical version of this claim is formulated by Bruno Latour, who writes, for example: “No one has ever seen a laboratory fact move outside unless the lab is first brought to bear on an ‘outside’ situation and that situation is transformed so that it fits laboratory prescription” (Latour, 1983, p. 166). For scientific knowledge to be useful in environments other than the artificial one in which it was developed, it is necessary to make those other environments more like the original artificial one in which the knowledge was created. Typically, this happens in the following way: the novel tools we introduce into the world to detect (e.g., a disease, climate change) or intervene (e.g., to cure a disease, to produce sustainable alternatives) require a set of conditions without which they cannot function. So if we want to use these tools in other environments, we must first intervene in those environments to ensure that these conditions are met.

A clear version of this argument is articulated by Hans Radder:

Experiments and technological projects do not need only to be theoretically described: they also need to be materially realized. Closed systems are nearly never ‘just found’. We practically always have to produce and maintain them through active intervention. The crucial point is that, to this end, theoretical-scientific or theoretical-technological knowledge plus experimental or technical know-how are surely necessary, but they are not sufficient. In order to be able to guarantee the required control of the relevant situations we need, at the same time, knowledge and possibilities of control of the *social* setting of the system. (Radder, 1986, p. 666, emphasis in the original)

For Radder, technologies should be evaluated not only in terms of their *effects*, but also in terms of their *conditions*: what is materially and socially necessary for a technology to work? To illustrate this, Radder uses the example of nuclear energy. As he argues, “nuclear energy, being a large-scale and complicated technology, demands extensive interventions from the central government.” (Radder, 1986, p. 669) These interventions are not only technical, but also political, e.g. “increased secrecy, intrusions into the privacy of workers and neighbours, restrictions on the freedom of demonstration, and the like.” (Radder, 1986, p. 669) As Winner (1980) had already argued, nuclear power is a technology that seems to work in its present form only when it is aligned with a centralized and, to some extent, anti-democratic form of government. This kind of political intervention is necessary to make the technology work. Nuclear energy systems require highly specialized expertise, stringent security measures, large-scale infrastructure, and long-term planning—all of which tend to concentrate power in the hands of a technical and bureaucratic elite, often beyond the reach of public deliberation or local control.

### Intervention as a political issue

If science is about representation, then the criterion of evaluation seems to be *accuracy*: does science describe the world accurately or not? But if science is mainly about intervention, then the criterion of evaluation becomes a matter of *appropriateness*: is science changing the world in an appropriate way? The next question, then, is what we mean by appropriate or inappropriate, or at least how we distinguish between the two. The simple answer seems to be that whether an intervention is appropriate or not depends on the values at stake. An intervention is appropriate if it helps us realize, or at least preserve, the set of values that we consider important and that we want to see embodied in the world.

This brings us to the question of values in science (Holman & Wilholt, 2022; Elliott, 2022). The values debate is predominantly framed in terms of the value-free ideal of science (VFI) and concerns the question: should non-epistemic values have an impact on scientific practices? While proponents of the VFI argue that non-epistemic values should be kept at bay, critics argue that (some) non-epistemic values can either play a positive role in epistemic practices, or at least cannot be avoided and should be recognized (see Longino, 1990; Douglas, 2009).

It is possible to see the revival of the philosophy of experimentation in the 1980s as a product of a version of this value debate. Many new experimentalists responded to sociological studies of science and experimentation (Pickering, 1984; Collins, 1985). These sociological studies formulated an argument against VFI: scientific debates can never be settled by internal epistemic values, typically because of some version of the underdetermination argument (e.g. Collins’ Experimenters’ Regress). Instead, they require external non-epistemic values to settle the discussion (e.g. social interests). Elliott (2022, 20) calls this the ‘gap argument’: “Evidential gaps between data and conclusions are inevitably filled by value-laden background assumptions, and thus, it does not make sense to exclude non-epistemic values from scientific reasoning.” In response, new experimentalists began to emphasize the role of experimentation in scientific practices in order to paint a more complex picture of the epistemic values involved in science (Franklin, 1986; Galison, 1987). Only within a narrow view of science, where logical deduction is the way to settle debates, is the sociological argument convincing. A focus on experimentation, however, shows that scientists often rely on a plurality of epistemic values to decide which theory they prefer.^5^

At the same time, reading the philosophy of experimentation as a mere instance of the value debate is too limited. As Ward (2021, 61) puts it, “[s]hoehorning everything related to science and the social into the literature on values in science has distorted the targets of investigation”. Philosophers of experimentation, for example, would object to what they call the representationalist idiom (see Pickering, 1995; Rouse, 1996) that is often still influential in the value debate, which understands scientific practices to be about producing accurate representations of the world. The political question is then what values, if any, should contribute to the production of these representations of the world.

Take the example of Heather Douglas, who is famous in the value debate for her formulation of the ‘inductive risk’ argument against the VFI: values come into play because scientists are always faced with levels of uncertainty, and thus have to make judgments about when it is acceptable to infer a hypothesis inductively from limited data. Her representationalism is shown in the way she defines science, namely as a pursuit aimed at “obtaining a more reliable, more true understanding of the world” (2009, 102). In this framework, the political consequences of science emerge downstream, when the empirical claims are taken up by policy-makers or the public. It is therefore not surprising that she frames the issue through the figure of the scientific advisor– the one who communicates claims to decision-makers– rather than through scientific practices themselves as sites of material intervention. Instead, the project focuses on how values should play a role once we consider the potential unintended consequences of making empirical claims that may be inaccurate or unreliable. “Given the public authority scientists wield, and the understanding of making empirical claims as a public action, there can be clear consequences for making a well-intended but still ultimately incorrect claim.” (Douglas, 2009, p. 72).

But when scientific practices are reconceptualized as intervening practices, the political question becomes different. A political philosophy of experimentation shifts attention from assessing the possible political consequences of empirical claims to the interventional nature of science, asking how experimental practices themselves produce consequences, distribute agency, and structure the world. As an alternative to the representationalist idiom, Andrew Pickering characterizes this perspective as the performative idiom:

Scientists, as human agents, manoeuvre in a field of material agency, constructing machines that, as I shall say, variously, capture, seduce, download, recruit, enrol, or materialize that agency, taming and domesticating it, putting it at our service, often in the accomplishment of tasks that are simply beyond the capacities of naked human minds and bodies, individually or collectively. (Pickering, 1995, p. 7)

One way of articulating this critique from a philosophy of experimentation perspective is through recent work on “materializing values” (Karakas & Tuboly, 2024). Their argument for looking at how values are present in the material culture of science comes close to the point raised here: “Instruments in science have a special characteristic, namely that they explicitly and clearly emerge from and remain embedded in social contexts, and are thus imbued with values.” (Karakas & Tuboly, 2024, p. 2) The project then becomes one of articulating a set of values that we want to see (materially) realized in society, coupled with an evaluation of scientific interventions in the light of these values.

Another connection is the critique articulated by Biddle and Kukla (2017, 217), who argue for a twofold shift in the focus of the literature on values in science: “from hypothesis acceptance or rejection to epistemic practices more generally, and from individual psychologies to knowledge-generating social institutions.” First, following Douglas (2009), the literature often interprets epistemic risk as ‘inductive risk’. However, most scientists’ judgments are of a more practical nature, e.g. when a cardiologist judges that there is an abnormality on the MRI scan, this is not an explicit hypothesis, but a practical judgment. In this sense, Biddle and Kukla prefer to speak of “phronetic risks,” echoing the Aristotelian notion of phronesis. Second, the literature on values in science focuses too narrowly on the individual psychology of the scientist. In reality, decisions are often made at an institutional level. The cardiologist has learned to judge abnormalities on scans because of institutional training. Even if the cardiologist wants to deviate from the institution, they may not have the authority or power to do so.

Both Karakas and Tuboly (2024) and Biddle and Kukla (2017) frame the question of values as an institutional and material one: it is not just a question of what the individual scientist believes, but of which interventions are possible within (the artificial environments introduced by) particular scientific practices. But both critiques still frame it as a question of *values*. A real political philosophy of experimentation should also address the question of *power* and *control*. Approaching scientific practice from these concepts involves more than just asking which (materialized) values influence decisions. It raises two further political questions: (a) Who has (or should have) the power to articulate any value that is even a candidate for consideration? (b) Who has (or should have) the power to decide which of these candidate values should actually be taken into account and which can be ignored? A political philosophy of experimentation should therefore not only ask which values are at stake, but also critique the existing institutions and power struggles that determine which actions are actually taken in scientific practices, and formulate proposals for procedures to do this better (according to articulated political values, e.g. justice, equality, etc.).

## Topics for a political philosophy of experimentation

The argument developed in the previous section leads us to the last step of the argument: the articulation of the political questions that philosophers of experimentation should address. If philosophy of science is no longer, not primarily, or not only about epistemological questions about science, what are the questions that philosophers of experimentation should be concerned with? I do not pretend to have an exhaustive answer to this question, but inspired by the previous historical overview, at least four clusters of questions can be distinguished.

### The moral evaluation of interventions

The first cluster concerns the moral evaluation of specific interventions. When the results of scientific practices lead to changes in society, these changes need to be evaluated. This area is typically associated with ethics and philosophy of technology. One could think of approaches such as Constructive Technology Assessment (CTA) (Rip, 1995), Value-Sensitive Design (VSD) (Friedman & Hendry, 2019) or Responsible Research and Innovation (RRI) (Burget et al., 2017). As those who are familiar with these approaches know, many of these issues are currently being taken up by a field such as Science and Technology Studies (STS), which is often concerned with critically mapping the ways in which technologies and scientific findings have been taken up in society. But again, there is no intrinsic reason why this could not also be part of the field of philosophy of science. We have seen, for example, how such evaluations are the bread and butter of Hottois’s bioethics committees and Ravetz’ extended peer communities.

So far, the reader may have seen how a philosophy of experimentation leads to ethics, but not yet how it leads to political philosophy. There are, therefore, at least three additional clusters of political-philosophical questions that emerge from this philosophy of experimentation.

### The politics of interaction

The second cluster is what I call the *politics of interaction*, and concerns how the interactions between scientific interventions and society should be politically organized. If scientific practices always intervene in society, then it is not just a matter of evaluating interventions on a case-by-case basis, but also of reflecting on the overall framework through which we structure these interventions. Inspired by Rouse (1987, 226–236), we can distinguish at least four types of questions in this cluster, related to (a) effects on (self-)understanding; (b) the proliferation of entities; (c) the extension of conditions; (d) the emergent network effects. Let me briefly discuss each of these in turn.

First, there are the *effects on (self-)understanding* associated with these transformations of society through science. This concerns the possible political consequences of the ways in which scientific knowledge can change the way we understand the world or ourselves. Here we can draw on some parts of philosophy of science that have already explored these political issues. We already discussed how, in the case of Douglas (2009), the question of non-epistemic values and the responsibility of the scientist was raised by the possible consequences of the uncertain empirical claims of the science advisor.

But another influential case under this banner is Kitcher (2001), who has explored the relation between science and democracy, arguing that a ‘well-ordered science’ should explore how “our attempts to achieve knowledge and the track records of their successes and failures, impinge on people’s values and interests” (Kitcher, 2001, p. 108). He therefore attempts to articulate a framework for assessing what research should be prioritized and argues that some forms of scientific research should not be funded (which is not the same as banning them), e.g. research into the relations between race and IQ. The political question here is how inaccurate or unreliable claims (e.g. about race and IQ) can lead to false beliefs about minorities and to harmful actions based on those beliefs. Inspired by John Rawls’ political philosophy, Kitcher argues that “we shouldn’t engage in ventures that can be expected to decrease the well-being of those who are already worse off than other members of society” (Kitcher, 2001, p. 98).

However, Kitcher’s work is both limited in its philosophy of science and its political philosophy. Kitcher’s philosophy of science focuses solely on the ways in which scientific theories shape people’s beliefs and indirectly contribute to harm when people act on those beliefs. A political philosophy of experimentation interprets these effects on (self-)understanding more broadly. It is not just that scientific practices can generate new models for understanding ourselves (“the brain is a computer”) and the world (“nature is an ecosystem service”). It is also about the ways in which these models are themselves embedded in our environment, making certain (self-)understandings more likely. For example, the many technologies with which we monitor the Earth mediate how we understand nature, for instance as something that can be quantified. Thus, it is not only the beliefs of scientists that shape our understanding, but also the way the world is mediated by previous scientific entities that we have introduced into it (see Verbeek, 2005; Karakas & Tuboly, 2024). This raises questions about the implications, conditions and desirability of these practices for our (self-)understanding.

Kitcher’s political philosophy is based on the liberalism of John Rawls (see Roth, 2003). However, as Rouse (1987) emphasizes, philosophy of science should also engage with other political philosophies. For instance, while a *liberal* critique of science focuses on the question of whether the power exercised by science is supported by individual consent, a *liberationist* critique asks whether the power exercised by science contributes to the structural oppression or emancipation of minorities (drawing on work from feminism and postcolonialism) (see Harding, 2011). In addition, Rouse points to what might be called a *non-intersectionist* political philosophy of science, inspired by Jürgen Habermas (1987) or Arendt (1958), which focuses more on the question of whether the power of science is not overstepping its bounds and colonizing the lifeworld. Finally, there is a *politics of local resistance*, inspired by Foucault (1975) and Heidegger (1962), in which political critique provides political tools to local struggles. In this tradition, general normative frameworks, such as Kitcher’s, are distrusted because they are seen as part of the problem. The intention here is not to endorse any particular political philosophy, but to emphasize the plurality of positions that exist. A political philosophy of experimentation should examine the material mediations of our (self-)understandings and remain open to the full range of available political philosophies.

The second set of questions concerns the *proliferation of new entities* beyond their laboratory conditions. These are questions about how we should deal with the *general* phenomenon of scientific practices producing new entities that can disrupt existing practices. It is a political question in the sense that it does not concern the evaluation of an individual case, but the question of whether and how scientific practices *in general* should be allowed to introduce new entities.

The simplest answer to this question is complete scientific freedom, allowing scientists to develop whatever applications they wish. Although such a libertarian position is conceivable, most positions suggest that this process should be constrained in some way. We can again refer to frameworks such as TA, VSD, or RRI, but now at a different level. The application of TA, VSD or RRI can be seen as ethics, but the design of these frameworks can be seen as part of the politics of interaction. At this level we can also locate Hottois’s plea for the guidance of technoscience by bioethics committees. These committees are decision-making systems which, he argues, are capable of recognizing a plurality of perspectives and whose decisions are always provisional and revisable in the light of new technological developments. In arguing for this, Hottois makes a political proposal on how to regulate the proliferation of new entities.

Third, there is the *extension of conditions* that often accompany these new entities. As we have seen, experimental and technical systems only work if the right social and material conditions are in place. This can raise political questions in at least two ways. First, there are questions about the *responsibility and desirability of maintaining* these conditions, as Hans Radder’s example of nuclear power illustrates. If nuclear power produces nuclear waste that will last for thousands of years, the question arises as to who will be responsible for managing this waste over such a long period. As Rouse (1987, 227) suggests, “[t]he stimulation, protection, regulation, utilization, and remedying of the effects of these translations of laboratory practices, materials, and procedures now occupy a large proportion of our overtly political activity.”

But there are also questions about the *inappropriate* extension of these conditions. This can be illustrated by the work of Nancy Cartwright, which focuses on the critique of the universal applicability of scientific theories and methods (see Cartwright, 1989). In her work on evidence-based policy, she argues against the naive belief that scientific findings can simply be extrapolated to new contexts (Cartwright & Hardie, 2012). For example, randomized control trials (RCTs), often heralded as the ‘gold standard’ of scientific evidence, are sometimes treated as universally applicable guides to policy decisions. However, Cartwright argues that the success of RCTs depends heavily on local conditions, including cultural, economic, and institutional factors. Similarly, her work on development economics highlights how scientific practices aimed at improving social outcomes, such as anti-poverty programs, often fail when implemented without regard to the specific, practical realities of the regions in which they are deployed (Cartwright, 2007). Both examples show that the failure to consider the contextual, material aspects of science is not just a methodological oversight, but a political problem.

The final set of questions relates to the *emergent network effects* of the proliferation of these entities and their conditions. Many of these entities and conditions are not independent of each other, but either require the presence or absence of other entities and conditions, leading to conflicts when they interact. In this context, Rouse (1987) refers to two likely scenarios. Inspired by Perrow (1984), this can lead to “tight coupling” with its associated “normal accidents”: the increased interaction between all these technical entities leads to increased complexity, with an increased likelihood of accidents, which are the inevitable product of an accumulation of failures, as Perrow’s analysis of the Challenger accident illustrates. This raises political questions about the preferred governance of these networks. In particular, it raises questions about which accidents are acceptable, since they are often impossible to avoid altogether.

Second, there are scenarios in which, in response to this increasing interdependence of technical artifacts, “the natural environment is artificially simplified, controlled and stripped of some of its own capacity for self-regulation and buffering.” (Rouse, 1987, p. 231) Rouse cites the example of the “green revolution” in agriculture. As new agricultural technologies moved out of the laboratory and into existing farming practices, it was obviously a game changer in the fight against hunger. However, the large-scale success of these technologies often required simplification and control, both at the material level (e.g., what crops or machinery should be used) and at the social level (e.g., what farmers could do with their fields and these crops). Such detailed discipline of land and people raises political questions about whether and how it should be done.

As Rouse (1987, 246) points out, both scenarios should not be framed simply in terms of how technology *determines* how people should act. Often there is a more subtle ‘modal’ redistribution of possibilities: although one may still be able to do many different things after the introduction of a technology, the landscape of what is possible, necessary or impossible has shifted. This is a political question about the range of choices we should have in our lives, even if nominally there are always alternatives.

### The politics of organization

The third cluster of issues I call the *politics of organization*. This concerns not so much the relationship between scientific practices and society, but the internal organization of scientific practices themselves. Again, this set of questions stems from a focus on experimentation. If scientific research is not just a matter of ideas, but is fundamentally dependent on the instruments and technologies that make scientific practices possible, this opens up the question of a socio-material history of scientific knowledge: how is scientific research conditioned by the underlying socio-material organization of science?

One could read the politics of organization as a translation of the traditional epistemological project into a new vocabulary. Traditionally, the epistemological project has been framed as a question of method: what methods guarantee accurate knowledge in science? Philosophers of experimentation transform this question into a similar but different one: since science is a social and interventional activity, it is fundamentally dependent on a set of social and material conditions. The question of method thus becomes a question of organization: what social structure do we need to enable science to produce reliable and appropriate interventions? Since this social structure is heavily dependent on instruments and technology, this question also becomes a socio-economic one: how should we think about the economy of science, particularly in relation to issues such as the funding of research or the accompanying reward system for scientists? The answers to these questions will shape the questions, the research programs and ultimately the intervention strategies available to science. The politics of organization thus informs the politics of interaction. I will, again, discuss three sets of questions, although they are all closely interwoven: questions about what *actors*, what *norms*, and what *structures* scientific practices should have.

The first group concerns which *actors* should be involved in scientific practices. This question is seen at work in Ravetz’ (1971) critique of the ‘industrialization of science’: as scientific communities grow in size and cost, they become dependent on external actors, such as the state or industry, to define their goals and assess their success. Without the appropriate tacit knowledge, these external actors risk undermining the internal procedures that make scientific practices so successful.

But it could be linked to more recent work, such as that of Naomi Oreskes and Erik M. Conway (2010) on the phenomenon of “merchants of doubt”: the ways in which industries strategically use the internal mechanisms of scientific practices to promote doubt about scientific consensus - such as in climate science - in order to avoid regulation. A genuine critique of climate denialism cannot focus on theory alone, but must engage with a political analysis of the organization of science and the role of industry in it. Similarly, one can point to debates about whether excluded parties should not have a say in scientific research, ranging from patient organizations (Rabeharisoa et al., 2014) to Do-It-Yourself biologists, who aim to conduct experimental biology outside university contexts (Simons, 2021; Eireiner, 2025).

The second group concerns the *norms* that scientific practices should follow. This is a political critique of the moral economy of science. This is often linked to questions about the increasing commodification of scientific practices and their changing incentive structure. Here we can draw on the work of Hans Radder, in particular the way in which he has used his theory of the “material realization” of science for a comprehensive critique of the commodification of science. In his work, he also seeks to rehabilitate the famous Mertonian norms of science in order to argue for a moral economy of science in function of the “common good” (Radder, 2019).

But we can also draw on the recent work of Steven Shapin, in particular his *The Scientific Life* (2008). Shapin traces the shift from the ideal of the disinterested scientist to that of the scientist-entrepreneur, whose work is increasingly tied to market imperatives and private-sector goals. The rise of the scientist-entrepreneur complicates traditional ideals of replication and openness, as commercial incentives encourage the withholding of proprietary methods and data, directly affecting the quality and transparency of scientific practices. The growing appeal of lucrative careers in AI companies, for example, raises concerns about the internal diversity of science: as talented researchers gravitate towards commercially driven fields, basic research in less profitable areas may be neglected.

The third group of questions in this cluster concerns a political interrogation of the *structures* of science. One example is Sabina Leonelli’s (2023) work on ‘open science’. Leonelli challenges the prevailing assumption that simply “sharing resources” is sufficient to achieve genuine openness in the scientific community. This critique is based on a philosophy of experimentation. What holds a scientific community together is not so much a set of shared theoretical beliefs, but what Leonelli calls ‘repertoires’: “the well-aligned assemblages of skills, behaviors, and material, social, and epistemic components that groups may use to practice certain kinds of science, and whose enactment affects the methods and results of research, including how groups practice and manage research and train newcomers.” (Ankeny & Leonelli, 2016, p. 20).

When applied to open science, this transforms the question from an epistemic one of access, to a political one of the infrastructure and organization of science: how can open science relate “to the existing diversity in epistemic practices utilized by different research communities around the globe– and, in turn, to the varying socio-political settings in which research takes place” (Leonelli, 2023, p. 5)? For example, centralized data infrastructures and standardized practices often privilege well-funded institutions in the Global North, marginalizing researchers in less resourced contexts whose local repertoires may not align with dominant paradigms. This dynamic risks perpetuating rather than breaking down hierarchies within the scientific community.

As an alternative, Leonelli argues for a framework that emphasizes the epistemic diversity of scientific practices, which she links to the problem of epistemic justice: “The more a system of practice is prepared to reconsider its own boundaries and demarcation strategies, thereby reassessing the extent to which it can incorporate diverse sources and viewpoints, the more that system will mitigate epistemic injustice, which in turn enhances the system’s ability to generate novel, reliable knowledge.” (Leonelli, 2023, p. 39) The goal of open science sharing practices is therefore not simply to share information, “but rather to facilitate human agency, whether in the form of reasoning (resulting in scientific explanations or theories) or interventions (producing methods and tools to interact with the world).” (Leonelli, 2023, p. 52).

### The philosopher as activist

The last cluster of questions concerns the *political actions of the philosopher.* This cluster is particularly raised by the cases of Hottois and Ravetz. We have seen how their work led not only to a political critique of scientific practices, but also to active political engagement. Hottois sat on the bioethics committees he argued were needed; Ravetz wrote the policy reports and engaged in the postnormal science that he hoped would lead to better science. Similarly, Sabina Leonelli has not only described the politics of open science, but has actively participated in initiatives to reform open science. At the same time, not all political philosophers of experimentation do this kind of work. Should political philosophers of experimentation be political only in their theoretical work, or should they also engage in activism?

Let me begin by clarifying what I mean by political activism. Activism goes beyond simply taking a stance in one’s theoretical work. Most political philosophers of experimentation do that. Activism points to something more. As Frazer (2023, 1259) summarizes: “Activists, as the term implies, are also actively engaged in a conflict in a way that is intended to make the outcome better align with the stand that they have taken, helping some parties to the conflict more than others.” Such activism need not involve radical or subversive activities against the status quo; activities to reform or improve existing practices also count as activism. Although there is an extensive literature on activism and academia (e.g. Maccarone, 2005; Hale, 2008; Ypi, 2012; Hoffman, 2021), most philosophers of science are unaware of these debates.^6^ If a political philosophy of experimentation makes sense, then philosophers of science need to take these debates into account.

In general, there are at least three groups of questions to consider. The first concerns *boundary work*: whether and in what sense is activism part of genuine philosophy? In other words, is what Hottois, Ravetz or Leonelli do *part of* their philosophical work, or rather something they do *apart from* their philosophical work? For example, if philosophy is understood as the business of formulating correct and true arguments, what and how can activism contribute to this activity? Similarly, from an activist point of view, one might wonder whether philosophers qua philosophers have anything specific to offer to these activities.

There are possible responses to this. On the first point, one could argue that philosophers have a duty to make their arguments heard and implemented, and that this may require activism. On the second point, one could argue that philosophers contribute to activism as experts in their field (but see Gesang, 2010). A more radical proposal is Brister and Frodeman’s (2020) plea for “field philosophy,” which links philosophy and activism by arguing that philosophers should derive their central questions from the field:

Field philosophers […] approach social, technical, and policy problems from the perspective of their collaborators, rather than starting with a framework that has been hammered out in the philosophical literature; their work results in knowledge or an intervention that is not primarily an academic philosophical publication; and they typically engage with collaborative partners in inquiry over a longer period of time (Brister, 2021, p. 394).

This may involve cases such as “bioethicists working with health care professionals to develop surgical protocols, environmental philosophers working with land managers to articulate priorities for wildland preservation, and logicians working with programmers on applications in computational mathematics.” (Brister, 2021, 393–394) Some of the examples we have discussed can therefore be described as ‘field philosophy of experimentation’.

Regardless of the answer to this first question, there is a second, *epistemic* question to consider: does activism enhance or undermine the quality of one’s philosophical work? Van der Vossen (2015, 2020), for example, has argued that political activism risks undermining the quality of political philosophers’ work. Once we align ourselves with a particular political cause, we risk becoming biased in favour of that cause and its advocates, with whom we identify, and thereby risk ignoring genuine critiques of our philosophical position. In response, some bite the bullet and argue that some forms of activism, such as climate change or world hunger, are worth the risk (Caney, 2012; Pogge & Cabrera, 2012). Others argue that a closer confrontation with the field is actually something that can improve the quality of arguments (Ypi, 2012; Hendrix, 2012). Finally, some argue that partisanship and objectivity are actually compatible if objectivity is understood as “engagement with inconvenient evidence” (Frazer, 2023).

To conclude, there is also the *normative* question of whether philosophers of experimentation *should* become activists. The “should” in this phrase is ambiguous. We have already discussed one version of it, namely whether philosophers have the *right* to become activists, or whether this undermines the quality of their work, either because one *does not want* to consider counter-arguments because of one’s allegiance to the cause (Van der Vossen, 2015; Vossen, 2020), or because one *is not allowed* to recognize counter-arguments because you do not bite the hand that feeds you (Hopper, 2013).

But there is also the question of whether philosophers have an *obligation* to be activists, i.e. whether one can only be a good philosopher if one’s work also involves activism. This seems to be suggested, for example, by the notion of “field philosophy” (Brister & Frodeman, 2020): if your philosophy is only relevant to other philosophers, that is problematic. Philosophy as field philosophy says that one should engage with the problems of non-philosophers, resulting in output that is relevant to their concerns. Should philosophers of science be concerned only with internal debates (e.g. about realism or the nature of causality) or should they rather take the problems from the actual scientific practices they engage with (Plaisance, 2020)? The latter seems to be at least part of the motivation behind the recent rise of the field of ‘philosophy of science in practice’ (see Ankeny et al., 2011).

A possible objection to this, however, is that philosophers have a poor track record as political activists, e.g. by supporting terrible political regimes (e.g. Lilla, 2001). But this is not in itself an argument against activism by philosophers. Pointing to (selective) examples of activism that, in retrospect, led to bad outcomes does not justify the conclusion that philosophers should never engage in activism. At best, it justifies that the question of the political role of the philosopher cannot be assumed, but should be the subject of discussion.

## Conclusion

This article has sketched out the contours of a political philosophy of experimentation. Using a wide range of examples, it has argued that such a political philosophy of experimentation already exists, but has often been rendered invisible. After systematizing the argument for a political philosophy of experimentation in Sect. 3, Sect. 4 tried to map out the questions that are central to a political philosophy of experimentation, in particular questions about the politics of interaction, the politics of organization and the political actions of the philosopher. In this paper I have not tried to take a particular position on any of these questions. My argument does not depend on whether one agrees with any of the specific diagnoses of the political situation of science discussed so far. Rather, the point is that these clusters of questions should become central once a focus on experimentation transforms philosophy of science into a political project.

Many scholars have already taken up these questions, but the question is whether they should also be part of the philosophy of science. What is at stake in this article, then, is also the identity of philosophy of science: what kind of project is philosophy of science? What are its central questions? And which authors and debates belong to the history of philosophy of science and which do not? In this sense, this article goes against a common historical narrative of philosophy of science, and of philosophy of experimentation in particular. The beginning of philosophy of experimentation is often located in the 1980s, associated with the work of new experimentalists such as Hacking (1983) or Galison (1987). However, this narrative has recently been problematized (Simons and Vagelli 2021; Potters & Simons, 2023). This scholarship shows that there is a longer history of philosophy of experimentation, such as Dingler (1928), Wind (1934) or Bachelard (1934), and that the debates of the 1980s contained more disagreement and plurality than is often recognized. What has happened, however, is a purification of history, in which only those philosophers of experimentation whose projects could be read in line with the dominant epistemological interests of Anglophone philosophy of science are remembered.

The argument in this paper supports these critiques in an additional way. My argument suggests that, like these older philosophers of experimentation, more recent political philosophers of experimentation tend to be excluded from philosophy of science because they do not share the epistemological goals that dominate much of contemporary philosophy of science. Their work is often no longer classified as philosophy of science, but rather as ethics, STS or philosophy of technology. Such classifications, however, are based on implicit preconceptions about the task of philosophy of science. There is no intrinsic reason why philosophy of science cannot be conceived as an essentially political project. In this sense, the question of whether philosophy of science should be political or not is not a question that can be answered by facts. Rather, it is a question of values that requires philosophers of science to take a stance on whether or not to undertake this political task.

## Data Availability

Not applicable.

## Footnotes

1 This raises an additional question, which I do not intend to address, namely, *why* are these authors omitted? Others have attempted to address this question. For example, following Susan Leigh Star (1992), Karakas and Tuboly (2024) suggest that it is partly due to a refusal on the part of philosophers of science to engage with the material dimension of science. This dimension is perceived as undermining the claims of scientific practices to be objective and universal (since material artifacts tend to be associated with situated and tacit knowledge).

2 Radder’s dissertation was originally published in Dutch in 1984, and an early English translation appeared in 1988. However, I use the recent, improved translation published in 2012 with an additional afterword by Radder.

3 The French quotes are translated by the author.

4 This epistemic argument has been particularly influential in STS, going back to a famous case described by Wynne (1989) about sheep farming after Chernobyl: scientists in the UK wrongly imposed rules on sheep farmers in Cumbria after the Chernobyl disaster, dismissing the local expertise of the farmers themselves. These ideas also echo many later calls to interpret the introduction of certain technologies into society as ‘collective experiments’ (Latour, 2011) or ‘social experiments’ (van de Poel, 2011). For example, proper testing of nuclear power or GMOs cannot be done in the laboratory alone, but must be done on a one-to-one scale in society. In this sense, these are experiments on society, which raises the question of whether stakeholders other than the scientists should have a say in the matter.

5 For a broader context of the rise of new experimentalism, see Potters and Simons (2023).

6 Though there are some internal debates on what ‘engaged’ philosophy of science looks like (see Plaisance & Elliott, 2021 ).
